# Benefits of effective diagnosis on impact of quality of life

**DOI:** 10.1186/2045-7022-1-S1-S75

**Published:** 2011-08-12

**Authors:** Anthony T Dubois

**Affiliations:** 1 Beatrix Children's Hospital, Department of Pediatric Pulmonology and Pediatric Allergy, Groningen, Netherlands

## 

Quality of life has become a central outcome measure in medicine over the last two decades as it is increasingly being recognized that quality of life methodology is the most appropriate outcome measure with which to answer the question: does a particular management strategy provide clinically meaningful benefits as perceived by the patient? While the first of such studies in the area of food allergy were qualitative in nature, validated instruments have recently been developed in the context of the Europrevall study which measure health-related quality of life (HRQL) in a specific and reliable way. Instruments are now available for adults, adolescents and younger children of all ages. These instruments allow researchers and clinicians to quantitatively assess management strategies for food allergy Despite the fact that there is no cure for food allergy at the present time, management of many patients is thought to result in reduced risk and hence improved HRQL. There are many aspects to the management of the food allergic patient, including accurate diagnosis, adequate patient education, and provision of emergency medication where appropriate. All these elements may contribute to HRQL changes seen in patients with food allergy. Studies on the effects of effective diagnosis in the form of (double blind, placebo controlled) food challenges show that HRQL improves in patients undergoing this procedure. Although this improvement is greater in patients in whom the allergy is shown to be absent than in patients in whom the food allergy is confirmed, all patients show improvement in HRQL after having undergone this test. Thus, effective diagnostic testing is important to patients as it has a positive effect on HRQL. This effect is probably the result of diminished uncertainty that patients have about their disease resulting from the test.

